# Cointegrations in house price dynamics and ageing population risks

**DOI:** 10.1371/journal.pone.0296991

**Published:** 2024-02-12

**Authors:** William M. Cheung

**Affiliations:** Waseda Business School, Faculty of Commerce, Waseda University, Tokyo, Japan; Royal Melbourne Institute of Technology, AUSTRALIA

## Abstract

How does the riskiness of an ageing population change with house price dynamics of rural areas? Why do rural house prices increase faster than cities despite their ageing populations? Life cycle theory predicts working age households have higher demand for housing than retirement households. An issue that has seen much less attention in the literature is that rural house prices have been increasing despite their populations age rapidly. To answer these issues, our paper introduces an empirical cointegration-based framework designed to be flexible for empirical settings. Our cointegration framework reveals crucial information about rural housing and ageing which has not been found previously: the short-term deviation of house prices from cointegration restrictions is a strong predictor of future rural house prices and migration rate from 1 to 4 year ahead. This is not the case for urban areas nor where cointegration restrictions are being ignored. Rural house prices, not urban ones, are the key to understand this cointegration restriction. Our framework is pertinent to most ageing societies with available housing and demographic data. When a government formulates macroprudential policies internalizing these cointegration restrictions and supporting rural developments, migration into rural areas and population increases are possible. Our evidence highlights the importance of cointegration-based long-run ageing risks for rural housing markets.

## 1. Introduction

How does the riskiness of an ageing population change with house price dynamics? Why do rural house prices increase faster than cities despite their ageing populations? Life-cycle theory of consumption [1, 2] and its extensions depict a negative relation between population age and house prices: with a decreasing population growth rate, there are more old people than young, more dissaving than saving, resulting in net negative saving in an economy. Dissaving reduces housing demand, hence house prices. While effects of demographic changes on housing prices are well documented in the literature, house prices also affect characteristics of a population through saving and consumption. Therefore causality and conclusions from the literature are mixed at best [3–11].

Drawing from the above, we model house prices and ageing population as a cointegration relation instead of a causal relation as in the literature. Our paper introduces an empirical framework designed to be flexible and extendable to settings in different countries. Unlike urban house prices, rural house price appreciations, which are not receiving much attention in the literature, are the key to understanding cointegration restrictions of house prices and ageing population. Our framework is pertinent to many ageing societies with available housing and demographic data. We illustrate its effectiveness by applying it to property transactions and population characteristics from an advanced economy, Taiwan, because of the longevity and consistency of its data. We emphasize that our framework can be easily modified to different market settings in ageing economies with serviceable data sources. We provide a section on implementation of our framework to more general settings pertinent to other ageing economies.

We suggest that the cointegration relation between house prices and ageing population, a measure of long-run ageing risk, is a missing and crucial factor of house price dynamics particularly for rural areas. Autocorrelations of cointegration residuals, from an error correction model, decrease rapidly, implying that the long-run dynamics of house prices and ageing population are connected by a common long-run component. Cointegration residuals account for significant variations in the growth of rural house prices in both short and long horizons. However, it is not the case for urban house price growth. Finally, the error correction term predicts migration rate positively (negatively) in rural areas (urban areas), suggesting more people moving-in rural areas than moving-out following a larger correction of house prices. Our results are robust to definitions of rurality and ageing. Overall, our findings suggest the importance of cointegration-based long-run ageing risks for housing markets of rural areas.

Our paper contributes to the literature in the following ways. First, house price dynamics in rural areas, where population aged faster than urban areas, are much less studied than urban ones. Previous studies cited earlier are either focusing on urban areas or country level. A rural housing market is important for rural households and rural economy is a pillar for our overall economic stability. We introduce an empirical framework, which is pertinent to many ageing societies with available housing and demographic data, to study the rural house prices and ageing population. We highlight the importance of long run ageing risks for rural housing markets. Contrary to current literature, long run ageing risks are insignificant predicting future urban house prices. Our second contribution is to provide an answer to why rural house prices increase faster than cities despite their ageing populations. When a government formulates macroprudential policies internalizing these cointegration restrictions and supporting rural developments, migration into rural areas and population increases are possible. To formulate rural rejuvenation policies, policy makers may support rural property redevelopment through creations of new business opportunities and attract young adults to return to their rural hometowns. This is consistent with goals of rejuvenation programs of other fast-ageing economies like Japan and Italy. From January 2023, the Japanese government is offering JPY 1 million (USD 7500) per child to families who move out of greater Tokyo area. In 2022, local villages of Italy, e.g. Santo Stefano di Sessanio, are offering EU residents up to USD 28500 for three years to move and work there. Our framework could be applied to these countries when their data sources are available.

Third, from an econometrician’s view, Taiwan provides an interesting setting to study house prices of rural and urban areas. The rural areas horizon-specific linkages between housing, ageing and macroeconomic variables we found in this paper, which are missing in earlier studies, are within the period of 2011 Rural Rejuvenation Act (RRA) of Taiwan. Details of the Rural Rejuvenation Act is available at the Laws and Regulations Database of the Ministry of Justice, Taiwan. The RRA was signed into effect in 2011 as one of the largest government-funded Rural Rejuvenation programs among advanced economies. For classification of economies, we refer to the metric from the World Bank Country and Lending Groups. Taiwan is one of 83 high-income economies (USD13,846 or more). The Farming Village Revitalization Act (2009) and Rural Community Revitalization Bill (2009) are part of the Taiwan government’s revitalization program. The Rural Rejuvenation Act was signed and announced on 4^th^ October 2010 by the Taiwan government. The Act has been extended to 2026 after the injection of US$210.2 million in 2021. Under the RRA, a fund of NT$150 billion (US$4.6 billion) over 10 years was established in 2012 by the Taiwan Ministry of Agriculture to finance development in rural communities, among the largest government-backed funding program of its type. By supporting rural development, Taiwan revitalized rural areas by bringing young adults back to their hometowns with new business opportunities, as reported by their Executive Yuan in 2018. Over 8000 local beautification projects were funded by the RRA during 2011 to 2020. Another NT$6 billion approved in June 2020 over 2021 to 2026. Taiwan is also one of the fastest ageing populations among advanced economies [12]. The revitalization of rural areas has been a part of the solution to the long run ageing risks of Taiwan and could be a useful reference for other ageing economies.

This paper is related to several studies in the literature. [4, 5] find no significant association between housing demand and price with UK and Japan data. [13] considers cointegration relation between US house prices and income and find no significant relation between them. [6] finds that ageing populations lower real house prices substantially with country level data from 22 OECD countries in a panel regression framework. [8] find that in Germany cities, house price appreciation is lower in cities that age more rapidly. They also find that house prices are cross-sectional dependent but panel stationary in first differences. [14] find that in a panel regression framework, ageing and house prices in Japan are negatively associated but unobserved in rural areas. [15] study ageing and house prices in Korea again in a panel regression framework but results between rural and urban areas are mixed. [16] find that house prices in China have a weak wealth effect on consumption but results vary across horizons. None of the papers cited above focus on long run ageing risk, measured by cointegration relation between rural area ageing and house prices. To our knowledge we are among the first to consider the impact of long run ageing risk on rural housing market and migration rates. We discuss in detail in the next section on related literature and positioning of this study with respect to them.

The rest of this paper is organized as follows. In Section 2 we review the literature. We describe our data and methodology in Section 3. Section 4 reports our results and discussion. Section 5 provides more discussions on implementations in more general settings. Section 6 concludes.

## 2. Related literature and contributions of this study

During the 20^th^ century, OECD countries such as Japan, Italy and Germany entered the ageing society due to the longer life expectancies and lower birth rates [17]. Population aged over 65 (80) is 17.3% (4.6%) in 2019, and 26.7% (9.8%) in 2050 by OECD projections (OECD 2021). The OECD defines a country as an aged society if the proportion of people aged 65 or older is between 15 and 20% and super-aged if this share is 21% or above.

Effects of demographic changes on housing prices are well documented in the literature [3, 9, 18–23]. House prices increase with baby boom and decrease with baby bust generation [3]. Their results suggest a linear positive relation between house prices and ratio of young adults to old adults. From 70s to 80s, baby boom entered housing market, ratio of young adults to old adults increases, house prices increase substantially. While the baby bust generation has not led to a substantial decline in house prices as predicted in [3], the demographic impact on house prices attracts wide attention from scholars and debates. Early evidence suggests that demographic changes have no statistically significant influence on housing prices in Canadian markets whose population composition is similar to the US data [4]. More recent studies find that demographics have a significant effect on housing prices through the short-run adjustment process in Japanese housing market [5]. Housing demand is also driven by human capital such as education, income, and health in all age [7].

On the other hand, studies suggest that housing prices affect household saving and consumption, both of which in turn are related to ageing population. In a dynamic overlapping generation model, [9] shows an increase in house price cause a substantial short-run decrease in saving as homeowners spend more their windfall gains or bequeath the additional housing capital to assist their adult child afford more expensive housing. [10] estimate empirically the household response of consumption to house prices. They estimate a large positive effect of house prices on consumption for the cohort of old households who are homeowners, and almost no effect for young households who are renters.

A more perplexing issue that has seen much less attention in the literature is that rural house prices have been increasing despite their populations age rapidly [24–26] (Cohen and Greaney, 2023; Department for Environment, Food and Rural Area UK, 2018a, b). In fact for the last two decades, rural house prices of “super-aged” economies such as Japan, Italy, South Korea, and China are far from decreasing [27]. Changes in house prices in rural areas bear significant consequences for an economy as they affect wealth of rural households and their ability to borrow [28, 29], as collateral [30, 31] and through consumption [10, 32]. Ability to finance activities of rural households is vital for sustainable development of an economy as they supply the foundation of economic growth through agriculture, forestry, and clean environments [33]. A steady growth of rural house prices is thus important to our economy. However, changes in house prices, especially in rural areas, are inherently difficult to measure, compared to stocks or bonds, because records of property transaction are not readily available and upon their availability, often with lags of several months.

This paper is closely related to several studies in the literature. Ohtake and Shintani [5] model housing demand and price with Japanese country-level data in cointegration framework but find no significant association, Takats [6] finds that ageing and house prices are negatively associated using data from 22 OECD countries in a panel regression framework. Hiller and Lerbs [8] find that in Germany cities, house price appreciation is lower in cities that age more rapidly. They also find that house prices are cross-sectional dependent but panel stationary in first differences. None of the papers cited here and in the introduction focus on rural ageing population and house prices, which is the focal point of this paper.

Drawing from conclusions of the literature, we model house prices and ageing population as a cointegration relation instead of a causal relation as in the literature. Our paper introduces a framework designed to be flexible and extendable to empirical settings for different countries. This framework is pertinent to most ageing societies with serviceable data of house prices and population. More importantly our conclusion of positive migration rates to rural areas is attributable to many ageing societies with rural revitalization programs and is not unique to where data is originated. As such, our paper contributes to the literature by introducing a cointegration-based framework for empirical analysis which uncovers policy-relevant information and is easy to implement or extend in various settings. This framework enables researchers to incorporate a variety of spatial and intertemporal information which goes beyond a solitary case study. This, in turn, adds significant value to the literature which focus on either urban area [3–5, 7, 8] or cross country with aggregated country level data [6].

Unlike urban house prices, rural house price appreciations, which are not receiving much attention in the literature, are the key to understanding cointegration restrictions of house prices and ageing population. Our framework is pertinent to many ageing societies with available housing and demographic data. When a government formulates macroprudential policies internalizing these cointegration restrictions and supporting rural developments, migration into rural areas and population increases are possible. Our evidence highlights the importance of cointegration-based long-run ageing risks for rural housing markets.

The positive impact of cointegration residuals to rural migration rates would be useful for policy makers from advanced economies with ageing populations beyond Taiwan. In order to formulate rural rejuvenation policies, policy makers may support rural property redevelopment through creations of new business opportunities and attract the young adults back to their rural hometowns. Finally, Taiwan provides a unique setting to study house prices of rural and urban areas. The horizon-specific linkages between housing, ageing and macroeconomic variables are supported financially by the 2011 Rural Rejuvenation Act (RRA). With the 2011 RRA, Taiwan significantly revitalizes rural areas while successfully cultivates young adults to go back to their hometowns with new business opportunities which could be a useful reference for other ageing economies.

## 3. Data and methodology

### 3.1. Taiwan housing market and its ageing population

Taiwan is an aged society as the proportion of population aged 65 or above is over 14% in 2018. It is estimated that in 2025, Taiwan will enter the era of super-aged society in which the population 65 aged accounts for over 20%. The speed of population ageing in Taiwan is 1.6, 2.9, and 7.3 times faster than in Japan, United States, and the United Kingdom, respectively. Rapid population ageing has significant implications on health care [34, 35], labor [36], and housing [37]. According to Taiwan’s Ministry of Interior, the ratio of house price to annual household income of Taipei rose from 6.4 in 2004 to 15 in 2018 which is comparable to Hong Kong with a score of 18, significantly higher than Singapore at 4.5 and Tokyo at 5.0. Relative to Taipei, the benchmark house price-to-income ratio of Taiwan is 9.2 in 2018. The price level of housing market in Taiwan is difficult for new generations to buy a house on their own.

Taiwan has been influenced by Chinese culture and Confucianism [38, 39]. In Confucius concept the basic unit of society is a family not an individual. Confucius rates people depending on how well they help their family instead of personal achievements [40]. It is possible that when young adults face difficulties of home ownership, parents may provide financial assistance including the down payment or direct financial transfer which increase significantly home ownership of young adults [41–43]. We posit that population ageing may have a positive effect on housing prices with proper incentives for young adults to return to their rural hometowns.

### 3.2. Data

Our housing transaction data is from the Department of Land Administration, Ministry of the Interior (MOI), Taiwan. This transaction data set is categorized into seven municipalities include Taipei, Kaohsiung, New Taipei, Taichung, Tainan, Keelung, Hsinchu City, and ten counties include Hsinchu County, Taoyuan, Hualien-Taitung, Ilan, Nantou, Pingtung, Changhua, Chiayi, Miaoli, and Yunlin. Chiayi includes Chiayi City and Chiayi County, Taichung includes Taichung City and Taichung County, Tainan includes Tainan City and Tainan County, Kaohsiung includes Kaohsiung City and Kaohsiung County, and Hualien-Taitung includes Hualien County and Taitung County. Our sample covers 90.5% of the GDP of Taiwan and 98.9% of Taiwan’s total population (Department of Statistics, MOI 2020). Following the literature in urban-rural studies [44–47], we use the percentage of population as a measure of rurality. Our results are qualitatively similar with alternative definitions of urban-rural areas such as the population density, distance to the nearest major metropolitan area, or special municipality of Ministry of Interior. Our sample of urban areas include Taipei, New Taipei, Taoyuan, Taichung, Tainan, and Kaohsiung. Rural areas include Changhua, Chiayi, Hsinchu City, Hsinchu County, Hualien-Taitung, Ilan, Keelung, Miaoli, Nantou, Pingtung, and Yunlin. Our set of indices is analogous to the S&P CoreLogic Case-Shiller 20-city indices. S2 Table of our Appendix reports our annual indices, and monthly indices are available in our internet appendix. Our sample period is from January 2012 to December 2018. Our sample excludes housing transactions in 2019 to reduce political uncertainty originated from elections on risk premia [48, 49] and 2020–2022 due to impact of COVID-19. For Hualien-Taitung, Ilan, Nantou, and Pingtung, housing transaction data is available only from June 2012 onwards. Our data set of transaction records is comprehensive, contains address, zip codes, the type of houses, the size of land, the floor, the total floor of the whole building, the size of building, transaction date, the layout (e.g., bedroom, hall, and bathroom), and the transaction price.

We use transaction data to construct house price indices of seventeen areas in Taiwan based on the repeat sales approaches [50–52] (Bailey et al, 1963; Case and Shiller, 1987, 1989). To simplify our representation of locations, our paper uses the term “areas” to represent counties and cities. We filter incomplete records, and transactions of commercial buildings and factories. We exclude records without prices or addresses, replicates, and zero-dollar transactions. As there are no predetermined, unique identifier for each property, we develop a naming system named “Home Code” to convert property level information in Chinese to a series of numbers and English alphabets. Home Code is a concatenated string with zip code, size of land, size of building, number of bedrooms, number of halls, number of bathrooms, the floor, indicator of a detached house (W) or condominium (F). Consider an apartment locates on the fifth floor of a building in Da’an District of Taipei city (zip code = 106) with three bedrooms, two halls, and one bathroom, with the size of land and the size of the building is 19.39 m^2^ and 100.98 m^2^, the Home Code of the house will be “1061939100983215F”. Once we identify each house individually, we count the number of sales for each house. Following the literature, we filter the houses only had one transaction record and remove the houses that were traded twice within two consecutive dates.

We decompose *n* multiple sales into *n-1* number of pairs of sales. If a house were traded three times, we consider the houses has the two independent pairs of trades sequentially. Our final sample consists of 221,290 observations, equivalent to 110,645 pairs of transactions. Table 1 reports the summary statistics of the housing transaction in seventeen cities of Taiwan. Consistent with expectation, Taipei has the highest average price per square metre at 146,862 NTD (4,800USD), followed by New Taipei at 82,892 NTD, Hsinchu County at 69,792 NTD (2,273USD), and Hsinchu City at 63,231 NTD (2,059USD). This is not surprising as Taipei is the capital of Taiwan, New Taipei is the second largest city of Taiwan, Hsinchu is the center of semiconductor industry, home to the TSMC, the world’s most valuable chip maker. Chiayi has the lowest price per square metre at 29,977 NTD (976USD), followed by Pingtung at 30,077 NTD (979USD), Hualien-Taitung at 33,792 NTD (1,100USD), and Keelung at 39,492 NTD (1,286USD).

**Table 1:** Summary statistics of housing market transactions.

Area	Number of transactions	House price (in NTD 1,000 per m^2^)			Change in house price index			
		Mean	S.D.	Median	P1	Mean	S.D.	P99
Changhua	3,348	46.662	31.992	40.000	-0.140	0.088	0.572	0.162
Chiayi	3,632	29.977	25.985	24.615	-0.189	0.100	0.796	0.227
Hsinchu County	8,646	69.792	50.662	61.538	-0.107	0.031	0.410	0.081
Hsinchu City	6,970	63.231	40.869	55.346	-0.107	0.035	0.297	0.077
Hualien-Taitung	4,616	33.792	28.615	26.923	-0.308	0.129	0.788	0.200
Ilan	4,418	43.315	30.754	36.154	-0.191	0.080	0.668	0.277
Kaohsiung	22,354	48.423	42.623	38.462	-0.167	0.064	0.440	0.151
Keelung	6,678	39.492	30.5	30.769	-0.166	0.070	0.652	0.237
Miaoli	3,456	42.977	25.708	40.000	-0.105	0.052	0.434	0.141
Nantou	1,424	41.085	28.869	33.077	-0.151	0.101	0.827	0.145
New Taipei	47,286	82.892	55.108	71.538	-0.116	0.014	0.307	0.056
Pingtung	3,914	30.077	26.677	21.538	-0.124	0.096	0.770	0.194
Taichung	44,038	61.831	63.777	52.069	-0.071	0.062	0.232	0.050
Tainan	13,892	46.708	39.415	37.308	-0.115	0.075	0.407	0.103
Taipei	13,306	146.862	159.977	106.769	-0.057	0.036	0.342	0.066
Taoyuan	31,322	54.585	29.923	49.731	-0.095	0.035	0.277	0.075
Yunlin	1,990	40.238	28.869	35.808	-0.096	0.074	0.489	0.085

This table reports number of property transactions, transaction price per square metre in NTD thousands, percentage changes of house price indices of seventeen major housing markets including Taipei, New Taipei, Taoyuan, Taichung, Tainan, Kaohsiung, Changhua, Keelung, Ilan, Miaoli, Nantou, Yunlin, Pingtung, Hualien-Taitung, Chiayi, Hsinchu City, Hsinchu County. Sample period is from January 2012 to July 2018 for all areas with the exceptions of June 2012 for Nantou and May 2012 for Hualien-Taitung. S.D. represents standard deviation. P1 and P99 represent the 1^st^ and 99^th^ percentile of monthly percentage changes. Means and standard deviations of percentage changes of house price indices are annualized.

For area-specific macroeconomic characteristics, we obtain the relevant data from the Department of Statistics, MOI Taiwan. Table 2 reports summary statistics for macroeconomic characteristics in our sample including unemployment rate, total population, population between 20 to 64, population above 64, and migration rates in our sample. For brevity, we report the summary of average statistics across areas. Further breakdowns of macroeconomic characteristics for each area from 2012 to 2018 are available in the Appendix.

**Table 2:** Summary statistics of macroeconomic characteristics.

	Panel B: Summary Statistics of Macroeconomic Characteristics		
	Mean	Standard Deviation	Median
unemployment rates (%)	4.115	0.52	3.95
total population (in millions)	1616.586	18.047	1620.560
population between 20 to 64	1066.497	10.060	1069.998
population above 65	227.205	25.441	228.610
migration rates	-0.174	0.404	-0.076
Number of guest houses	1012.5	364	985.5
Total housing units	590,676	12100.5	588,136

This table reports summary statistics of macroeconomic variables across seventeen areas. Unemployment rate is population aged over 15 without employment including job hunters, scaled by total population. Total population, population between 20 to 64, population above 65 are in millions. Migration rate is log difference between population moving in and moving out for each area. Number of guest houses is the average number of Class-4 properties defined by the Taiwan Tourism Bureau. Total housing units is the average of residential units. Sample period is from January 2012 to July 2018 for all areas with the exceptions of June 2012 for Nantou and May 2012 for Hualien-Taitung.

Average unemployment rate is at 4.1% with standard deviation of 0.52%, close to Malaysia, South Korea, and China [53]. Total population is about 1.62 billion, within this population between 20 to 64 is 1.07 billion, and above 65 is 227.2 million. Population displays moderate variations with a steady decrease in young people and increase in elderly over 65 from 11.15% in 2012, 14.56% in 2018 to 17.56% in 2022 (Ministry of Interior, 2022).

### 3.3. Methodology

#### 3.3.1. Repeat-sales approaches

There are two key constraints in implementing the repeat sales approach [50, 54]. First, only the transactions of the same house at different time stamps are included in the sample. The repeat sales method is a popular choice in the literature and among practitioners plausibly because only prices, sales date, and address of properties are needed for its construction. House price indices such as the Standard and Poor’s/Case-Shiller Home Price Indices in the US and house price indices in the UK and Australia, were built by the repeat-sales method. Second, it is more difficult to construct house price indices in areas which property markets are not active.

Shiller [54] develops the arithmetic repeat sales method (ARS). His paper argues that the arithmetic repeat sales method should be used instead of a geometric repeat sales method (GRS). First, the value of assets is related to arithmetic averages of price, not the geometric one and it is more naturally analogous to other indices, such as stock price indices. Second, an arithmetic index is much less affected by those observations which are sold for a much lower price than the market price, say zero or one dollar. In other words, if a house is sold for one dollar, it will have a significant impact on a geometric index, but not on an arithmetic index. In additions, the value-weighted arithmetic repeat sales method (VW-ARS) gives more weight to the more valuable houses, the HPI constructed by the VW-ARS method is more influenced by the rise of the more valuable houses. However, several disadvantages of the repeat-sales methods as [55]: (1) it does not separate house price change from depreciation, (2) renovation between sales is ignored, (3) the sample is not representative of the stock of housing, (4) attribute prices may change over time, and (5) many sales are required before a reasonable repeat-sales sample is obtained. Shimizu, et al. (2010) improve This methodology could be improved by take net depreciation into computation [56]. We first illustrate the index construction using VW-ARS and GRS method. Then we discuss repeat sales methods and other index construction methods in the section after.

We estimate the value-weighted arithmetic repeat sales (VW-ARS) method from the geometric repeat sales (GRS) method and to build house price indices in Taiwan. We start with the constraint that each house must have been traded twice. Our input variables include home Code, Transaction date, and Price. Next, we estimate an index ***ζ*** of log house prices by estimating the following regression (in its general form):

y=Xζ+ϵ (1)

$$y={\left(y_{1},\ldots ,y_{n}\right)}^{\prime }$$ (2)

$$X=\left(\begin{array}{ccc} x_{11} & \cdots & x_{1T}\\ \vdots & \ddots & \vdots \\ x_{n1} & \cdots & x_{nT} \end{array}\right)$$ (3)

where definitions of ***y*** and *X* are different for GRS and VW-ARS, denoted as ***y***^***GRS***^, ***X***^***GRS***^, and ***y***^***ARS***^ and ***X***^***ARS***^. For GRS $y_{i}^{GRS}$ equals ln(*P*_*ik*_)−ln(*P*_*ih*_) where *P*_*ih*_ is the price of the first transaction of house *i* during period *h*, and with *P*_*ik*_ is the price of the second transaction of house *i* during period *k*. For VW-ARS, $y_{i}^{ARS}$ equals *P*_*i*0_ if the first sale of house *i* is in period 0, and zero otherwise. For GRS $x_{ij}^{GRS}$ equals -1 if the time of the first sale of house *i* was *j*, 1 if the time of the second sale of house *i* was *j*, equals zero otherwise. For VW-ARS, $x_{ij}^{ARS}$ equals -1 multiply by the price of the first sale of house *i* if the time of the first sale was *j*, the price of the second sale of house *i* if the time of the second sale was *j*, equals zero otherwise. We summarize the methodological differences in S1 Table of Appendix.

To obtain a geometric house price index from the log price index ***ζ*** for time *i*, the *i-th* element of the ordinary least-squares regression coefficient vector

$$\zeta ={\left({{(X}^{GRS})}^{\prime }X^{GRS}\right)}^{-1}{{(X}^{GRS})}^{\prime }y$$ (4)

one ought to compute the exponential function of vector ***ζ*** and multiply 100. Note that ***ζ*** is a column vector of the log price indices from period 1 to period T.

For our paper, we employ the VW-ARS as in [54] and estimate the following:

$$\hat{\eta }={\left({{(X}^{GRS})}^{\prime }X^{ARS}\right)}^{-1}{{(X}^{GRS})}^{\prime }y$$ (5)

which is an instrumented reciprocal of the usual candidate of ordinary least square regression coefficient $\zeta ={\left({{(X}^{ARS})}^{\prime }X^{ARS}\right)}^{-1}{{(X}^{ARS})}^{\prime }y$, which has an error in variable problem arises from stochastic independent variables. To get the VW-ARS in period *i*, we compute the inverse of ${\hat{\eta_{i}}}^{-1}$and multiply it by 100.

**Fig 1:**
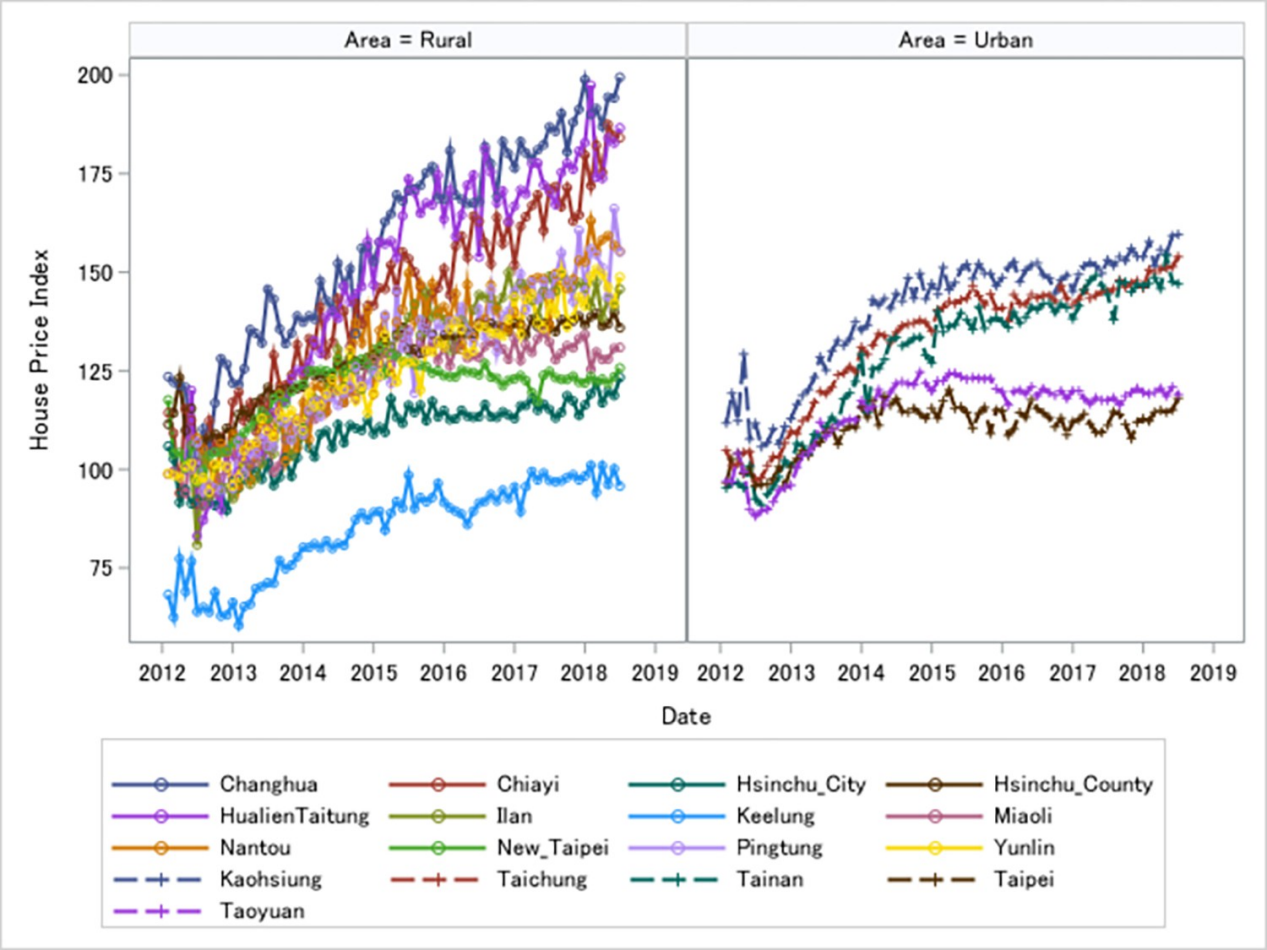
House prices of rural (eastern and midwestern) and urban areas (northern). Authors’ own visualization based on data by National Statistics, Ministry of the Interior (MOI), Taiwan (for property transaction prices).

We build a set of monthly and annual Case-Shiller style house price indices (HPI) for all seventeen prefectures including all rural and urban areas in Taiwan using over 1.5 million transactions from 2012 to 2018. Fig 1 illustrates the magnitude of price changes of house price indices. Figure on the left with solid lines and circles represents the dynamics of house price indices in 11 rural areas. Figure on the right with dotted lines and dashes shows the dynamics of house price indices in 6 urban areas. Contrary to conventional wisdom, within our sample period house prices increase more rapidly in rural areas than urban areas. Our visualization confirms with recent surge of rural house prices, similar to that in UK and France.

Table 1 reports the summary statistics of percentage changes of house price indices (HPIs). The annualized mean percentage change of HPI is the largest in rural areas, from 70% to 90%. Urban areas have the smallest annualized mean percentage change of HPI around 1% to 3%. Volatilities of HPIs, measured by their standard deviations, are highest at the eastern and mid-western rural areas, and lowest at the northern urban areas. We further examine time series properties of our HPIs in Section 4.

#### 3.3.2. Alternative house price index methodologies

The average sales method and the median sales method are simple ways to estimate a house price index. However, each property has its own characteristics and biases are hardly eliminated when using the average sales method. For example, when an extremely high-priced villa is sold, the average price increases and deviates temporarily from fundamentals. The median sales method published by the National Association of Realtors could be less susceptible to the influence of outliers, but still not accounted for property characteristics. Therefore, complex estimations have been developed to reduce biases.

**Fig 2:**
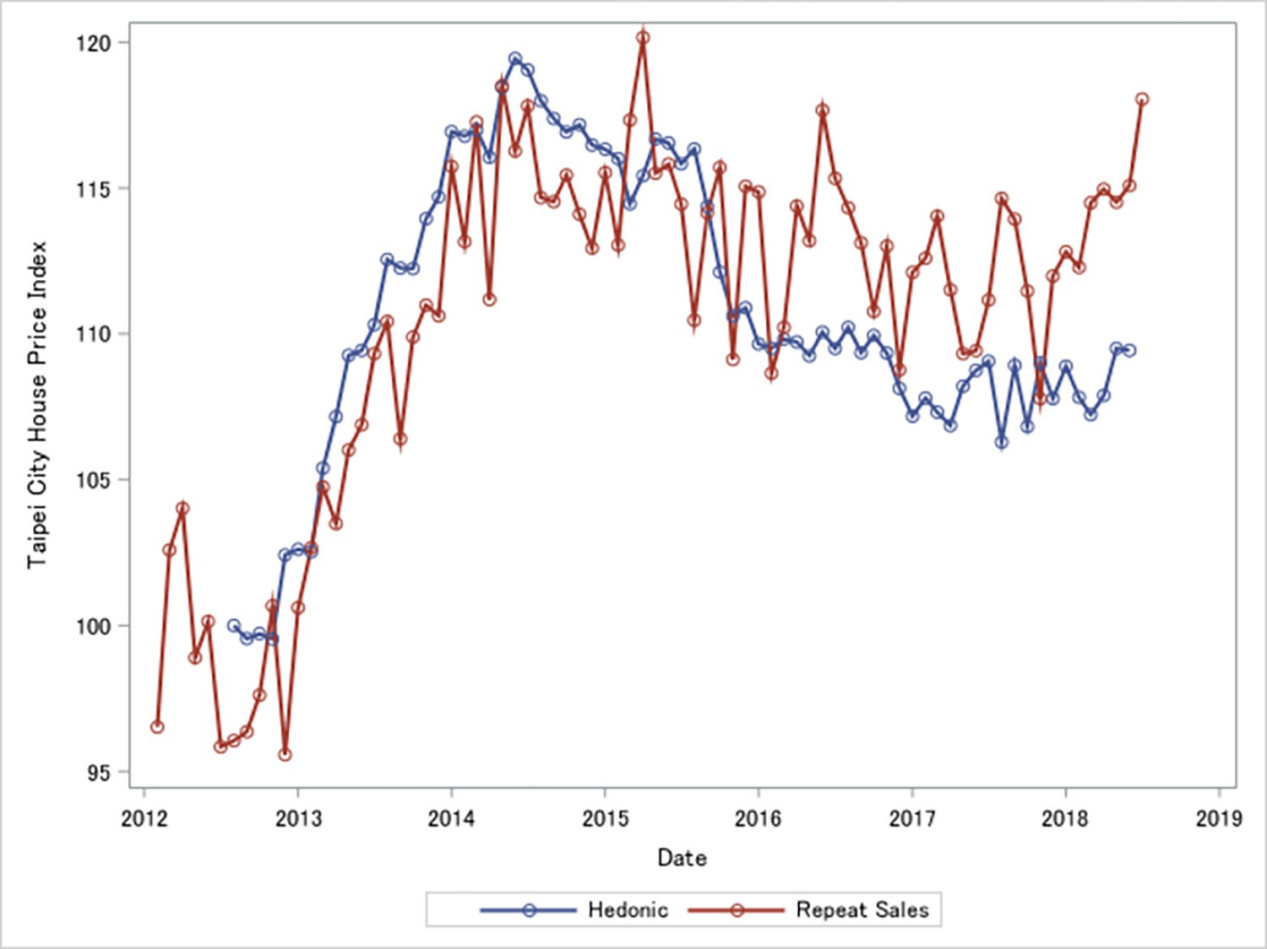
Repeat sales house price index and Taipei government hedonic index.

In addition to the repeat-sales approach, the hedonic method is also a well-known method for housing prices [57–59]. Hedonic pricing is widely used to construct home price indices globally, for example Halifax HPI of United Kingdom, and Japan Residential Property Price Index (JRPPI) by Ministry of Land, Infrastructure, Transport and Tourism. More discussion of the hedonic pricing literature in [60]. Hedonic pricing models assume house values are influenced by characteristics of houses in three categories including structural characteristics (e.g., size of the house, materials of construction), neighborhood attributes (e.g., distance to the downtown, facilities nearby), and environmental characteristics (e.g., risk of the natural disaster). However, [61] indicated two main disadvantages of the hedonic method. First is on the heterogeneity of housing market segmentations. Second is the misspecification of variables in the hedonic model. It is an empirical question on how to choose appropriate housing characteristics. The Ministry of Interior (MOI) of Taiwan government uses the hedonic method to construct housing price indices for six largest areas of Taiwan. We illustrate the differences between our repeat sales index and the MOI hedonic index in Fig 2. Long-run trends between our HPIs and MOI-HPIs are diverging especially after 2015. This difference suggests that our HPIs are more sensitive to changes of the housing market. Explanations of the differences between HPIs could be an important avenue for future house price index research beyond the scope of this paper.

## 4. Empirical results

### 4.1. Unit roots and stationary properties of HPIs

We employ standard analysis of time series properties of our set of area-based house price indices (HPIs) of Taiwan. We first examine unit roots and stationary properties of the HPIs with [62] unit root test of a house price index level. For each area, we regress change in HPI on lagged 1-period HPI, and 12 lagged changes in HPI, which can be written as $\Delta hpi_{j,t}=\phi hpi_{j,t-1}+\sum_{k=1}^{12}\mu_{j,t-k}\Delta hpi_{j,t-k}+\epsilon_{j,t}$ where *hpi*_*j*,*t*_ equals *η*_*jt*_ in (5) for VW-ARS, the house price index of area *j* at period *t*. We add a twelfth lag in the ADF tests to improve the fit of the model as in [63]. Table 3 reports county/city-specific coefficient of $\hat{\phi }$.

**Table 3:** Unit roots and stationary properties of county/city-level house price indices of Taiwan.

Rural area	$\hat{\phi }:hpi∼I(0)$		$\hat{\phi }:hpi∼I(4)$	
Changhua	-0.051	(-1.290)	0.664	(2.740)
Chiayi	-0.041	(-0.800)	0.659*	(5.570)
Hsinchu City	-0.135	(-2.030)	0.939*	(10.630)
Hsinchu County	-0.023	(-0.720)	0.505	(3.220)
Hualien-Taitung	-0.114	(-2.290)	0.598	(2.120)
Ilan	-0.111	(-2.300)	0.521	(2.370)
Keelung	-0.118	(-2.470)	0.882*	(13.680)
Miaoli	-0.053	(-1.640)	0.979*	(4.420)
Nantou	-0.053	(-0.840)	0.522	(2.160)
Pingtung	-0.085	(-1.520)	0.259	(0.990)
Yunlin	-0.031	(-0.690)	1.015*	(4.930)
Urban area				
Taipei	-0.279	(-2.750)	0.828*	(8.890)
Taoyuan	-0.059	(-2.150)	0.731*	(5.740)
New Taipei	-0.148	(-2.350)	0.820*	(9.320)
Taichung	-0.042	(-1.900)	0.906*	(12.670)
Tainan	-0.050	(-1.480)	0.863*	(8.360)
Kaohsiung	-0.041	(-1.190)	0.780*	(4.420)

This table reports county/city-specific coefficient of the Dickey and Fuller (1981) unit root test of a house price index level: $\Delta hpi_{j,t}=\phi hpi_{j,t-1}+\sum_{k=1}^{12}\mu_{j,t-k}\Delta hpi_{j,t-k}+\epsilon_{j,t}$. *hpi*_*j*,*t*_ is the house price index of area *j* at period *t*, $\Delta {hpi}_{jt}=hpi_{jt}-hpi_{jt-1}$. Column (1) report the coefficient estimate $\hat{\phi }$ of *hpi*_*j*,*t*−1_ the coefficient of the Dickey Fuller (1981) unit root test at a house price index level, *hpi*~*I*(0). Column (1) reports the coefficient estimate $\hat{\phi }$ of *hpi*_*j*,*t*−1_ the coefficient of the Dickey Fuller (1981) unit root test the fourth difference of a house price index, *hpi*~*I*(4). t-statistics are reported in parentheses. ***, **, and * represent 1%, 5%, and 10% significance, respectively. * indicates unit root test statistics that exceed 10% MacKinnon (1996) critical value of −3.59.

We report the estimates of $\hat{\phi }$ and their t-statistics at the index level under Column (1) and at the 4-th difference of a house price index under Column 2. t-statistics are reported in parentheses. We use confidence intervals from [63] for the Augmented Dickey-Fuller test. Their values are given as [-3.54, 3.54], [-2.91, 2.91], and [-2.59, 2.59] at 1%, 5%, and 10% significant level. We find that $\hat{\phi }$ of all HPIs are negative and smaller than the ADF statistics, implying all HPIs are nonstationary at their index levels. We further perform unit root tests at the fourth difference of HPIs. We find that $\hat{\phi }$ of 13 out of 17 HPIs are significant, implying they are stationary of order 4, denoted as *I(4)*. All 4 exceptions are rural areas including Hualien-Taitung, Ilan, Nantou and Pingtung. This is consistent with the asymmetric increases of HPIs in rural less populated areas.

### 4.2. Cointegration of house prices and ageing population

While each HPI is nonstationary separately, a linear combination of them with ageing population could be stationary, implying a possible cointegrating structure. As described in Sections 1 and 2, life-cycle consumption theory can be useful to describe a long-run relation between house prices and ageing population through aggregate saving [1–3] and consumption choices [9, 10]. With the life-cycle theory as the theoretical underpinning of the long run relation between house prices and ageing, we estimate their cointegration following the Engle and Granger [63] two-step procedure:

$$hpi_{jt}=\alpha +\beta \Delta Aged65+\nu_{jt}$$ (6)

$$\Delta \hat{\nu_{jt}}=\phi \hat{\nu_{jt-1}}+\sum_{k=1}^{12}\mu_{j,t-k}\Delta \hat{\nu_{jt-k}}+\epsilon_{j,t}$$ (7)

where *hpi*_*j*,*t*_ equals *ζ*_*jt*_ in (4) for GRS or *η*_*jt*_ in (5) for VW-ARS, the house price index of area j at period t. Δ*Aged*65 is the change in old-age dependency ratio, defined as the number of persons aged 65 years or above relative to number of persons aged 20 to 64 years. Eq (6) represents a linear, reduced form of housing-ageing association in the long-run implied by life-cycle consumption theory. We denote $\Delta \hat{\nu_{jt}}$ as the residual of (6), equals to $\hat{\nu_{jt}}-\hat{\nu_{jt-1}}$. In the ADF test in (7), we regress change in residual $\Delta \hat{\nu_{jt}}$ to the first lag of the residual $\hat{\nu_{jt-1}}$, and twelfth lags of the dependent variable in the ADF tests. We use 12 lags because we have monthly house price indices. Changing the number of lags does not affect our conclusion qualitatively.

Table 4 reports point estimate of cointegration parameters between house prices and ageing, sample autocorrelation functions of cointegrating residuals, and unit root tests of the stationarity of the cointegrating residuals. First, we note that for both rural and urban areas, sample autocorrelations of the cointegrating residuals are decreasing rapidly. This supports our view that dynamics of house prices and ageing population are guided by the same permanent component that can be eliminated by the appropriate differencing. The estimated cointegration parameters and standard deviations are reported in the first column. Cointegration parameters of both rural and urban areas are significant, suggesting house price fluctuations are associated with ageing population. The estimated cointegration parameter of rural areas is much larger than that of urban areas, suggesting house prices in rural areas have larger exposures to ageing risks than house prices in urban areas. Finally, unit root tests suggest cointegration in rural areas with test statistics -3.99 larger than MacKinnon [64] 10% critical value of -3.59. Collectively our results suggest that house prices and ageing population are cointegrated and they are difference stationary.

**Table 4:** Cointegration, autocorrelations, and error correction parameters.

	$\hat{\beta }$	ACF(1)	ACF(2)	ACF(3)	ACF(4)	Unit root test	Error correction $\hat{\gamma_{1}}$
Rural area	375.880*** (64.537)	0.736***	0.265*	0.162	-0.006	-3.99*	-0.613*** (0.111)
Urban area	211.548*** (50.720)	0.385**	-0.069	-0.059	-0.065	-2.03	-0.706*** (0.173)

This table reports estimates of cointegration parameters for rural and urban areas of Taiwan using the standard two-step procedure of Engle and Granger (1987). Cointegration parameters are estimated by regressing the detrended level of house price index on the detrended log change of old-age dependency ratio: $hpi_{jt}=\alpha +\beta \Delta Aged65+\nu_{jt}$. Old-age dependency ratio, *Aged*65, is defined as the number of persons aged 65 years or above relative to number of persons aged 20 to 64 years. House price index, *hpi*, is the VW-ARS in (5). Column $\hat{\beta }$ reports the cointegration parameters and standard deviations in paratheses. ***, **, and * represent 1%, 5%, and 10% significance, respectively. Columns labeled “ACF” report sample autocorrelation function of the cointegrating residuals for lags 1, 2, 3, and 4. ***, **, and * represent probability values for the correlations are 1%, 5%, and 10% significance, respectively. Column labeled “Unit root test” presents unit root tests for null that the cointegration residuals, $\hat{\nu_{jt}}$, contain a unit root: $\Delta \hat{\nu_{jt}}=\phi \hat{\nu_{jt-1}}+\sum_{k=1}^{12}\mu_{j,t-k}\Delta \hat{\nu_{jt-k}}+\epsilon_{j,t}$, with $\Delta \hat{\nu_{jt}}=\hat{\nu_{jt}}-\hat{\nu_{jt-1}}$. * indicates unit root test statistics that exceed 10% MacKinnon critical value of −3.59. The last column reports the adjustment speed, $\hat{\gamma_{1}}$, of short-run deviations of county/city-specific house price index to the long-run equilibrium in an error correction model: $\Delta hpi_{jt}=\gamma_{0}\Delta Aged65+\gamma_{1}\hat{\nu_{jt}}+\epsilon_{j,t}$. ***, **, and * represent 1%, 5%, and 10% significance, respectively.

### 4.3. Error correction model

Last part of the time series analysis of our HPIs is their error correction mechanism. We examine the speed of adjustment of short-run deviations to the long-run equilibrium between individual HPIs and Taiwan housing market by an error correction model:

$$\Delta hpi_{jt}=\gamma_{0}\Delta Aged65+\gamma_{1}\hat{\nu_{jt}}+\epsilon_{j,t}$$ (8)

Where our focus is $\hat{\gamma_{1}}$, the estimated coefficient of the error correction, which indicates the speed of which a house price index returns to equilibrium. A significant negative $\hat{\gamma_{1}}$ represents house prices converge to the long-run relation with ageing as in (6). Results are reported in the last column of Table 4. We find that both rural and urban areas have negative and significant $\hat{\gamma_{1}}$, suggest that corrections of short-run deviations of house prices from their long-run relation with ageing in albeit slowly. Overall, our time series results suggest that house prices in rural areas are difference-stationary, cointegrated with ageing and converge faster to long-run equilibrium with ageing upon short-run pricing deviations.

### 4.4. House price growth predictability of ageing risk

As depicted in the above, cointegration implies that house price growth rates are predicted by the cointegrating residuals. Current deviations of house prices from their long-run relation with ageing should forecast the dynamics of house price growth while house prices are converging to the long-run equilibrium. If the current house prices are exceptionally high, house price growth is expected to fall for house prices to adjust to the stochastic trend in ageing. As evident in the last section, cointegration in rural areas is more significant than in urban areas, therefore, we expect cointegrating residuals account for the larger variation in expected future house price growth in rural areas than urban areas.

We take advantage of the ability of our error correction specification to predict future house price growth at different horizons. In order to emphasize the importance of the cointegration relation of house prices and ageing, we compare values of the adjusted R^2^ for house price growth implied by the error correction model in (8) with the adjusted R^2^ values from a growth rate specification, that is (8) without the cointegration residuals.

We report results for house price growth in Table 5. We study our results at one-, two-, three-, and four-year horizons for both rural and urban areas. At the one-year horizon for rural areas, the adjusted R^2^ for the error correction specification is 0.549, compared to 0.305 for the growth rate specification without cointegration residuals. At longer horizons, the inclusion of the cointegration residuals becomes less important. At the 4-year horizon for rural areas, the adjusted R2 for the error correction specification is 0.899, compared to 0.700 for the growth rate specification without cointegration residuals. This is consistent with our model as the cointegration residuals capture transitory variation in house price growth rates. Both specifications in all horizons have significant model F-statistics, suggesting a strong fitness of our model in predicting house price growth. Next we turn to our results for urban areas. The differences between our results for urban areas and rural areas are stark. First, at one-year horizon, the adjusted R^2^ for the error correction specification for urban areas is 0.207, compared to 0.549 for rural areas. At all other horizons, the adjusted R^2^ for the error correction specification are negative, suggesting cointegration residuals do not account for house price growth in urban areas except for one-year horizon. At all horizons, the adjusted R^2^ for growth rate specification without cointegration residuals are close to zero with insignificant model F-statistics. These results suggest that change in ageing population alone does not account for house price growth in urban areas.

**Table 5:** Predictability evidence: House price growth.

House Price Growth Projection	Rural Areas		Urban Areas	
	With cointegration residuals	Without cointegration residuals	With cointegration residuals	Without cointegration residuals
*hpi*_*jt*+1_	0.549 (27.80***)	0.305 (25.12***)	0.207 (4.12**)	0.027 (1.83)
*hpi*_*jt*+2_	0.800 (66.99***)	0.437 (35.20***)	-0.122 (0.02)	0.006(1.13)
*hpi*_*jt*+3_	0.879(80.73***)	0.568 (44.36***)	-0.172(0.12)	0.000 (1.01)
*hpi*_*jt*+4_	0.899 (50.17***)	0.700(52.35***)	-0.100 (0.73)	0.104 (2.39)

This table reports the adjusted *R*^*2*^ for house price growth projections implied by the error correction autoregressive specification with cointegration residuals ($hpi_{jt+i}-hpi_{jt}=\gamma_{0}\Delta Aged65+\gamma_{1}\hat{\nu_{jt}}+\epsilon_{j,t}$) and the growth-rate model without cointegration residuals ($hpi_{jt+i}-hpi_{jt}=\gamma_{0}\Delta Aged65_{t}+\epsilon_{j,t}$) for rural and urban areas. One-year to four-year projections of house price growth are reported.

**Fig 3:**
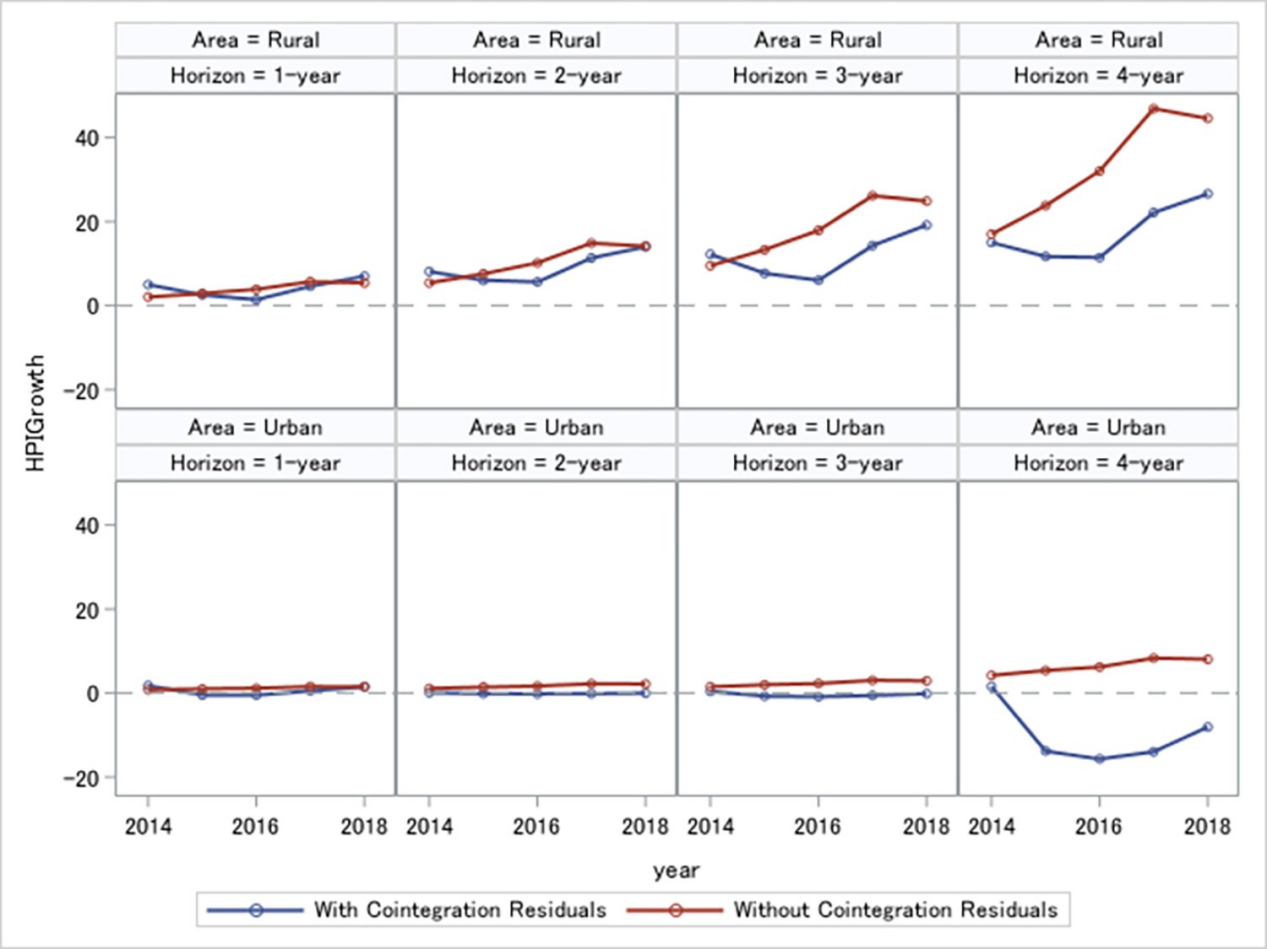
House price growth forecasts. This figure plots 1- to 4-year house price growth predicted by the error correction model and the alternative growth rate model. The latter model ignores the cointegration residuals in predicting future house price growth. House price growth is plotted for rural and urban areas.

The key takeaway of this section is that excluding cointegration residuals would significantly lower the predictability of house price growth and tweaks the conditional mean of house price growth. Fig 3 illustrates this critical point. We plot 1- to 4-year forecast of house price growth by the error correction specification (in blue), and the forecasts by the alternative, the growth rate specification (in red). Predicted conditional means are shown for rural areas (on the upper row) and urban areas (on the bottom row). We observe that the two specifications produce different predictions for future house price growth and are starker at longer horizons. This further reinforces our central idea that the cointegration residuals in our error correction specification contain distinct and valuable information about future house price growth beyond that in the growth rate specification.

Overall, results of this section highlight the importance of cointegration in the measurement of ageing risk of house price growth. Short term deviations of house prices from the permanent component in ageing–the cointegration residuals–contain valuable information for predicting house prices and their growth. Thus, it could represent a key component in the migration rate as well. We turn to this possibility in the section 4.5 and study how the cointegration residuals affect migration rate in rural and urban areas in different horizons.

### 4.5. Migration rate predictability of ageing risk

A key objective of the 2011 Rural Regeneration Act is to bring young adults back to their rural hometowns. We posit that the cointegration residuals, short term deviations of house prices from the permanent component in ageing, may contain valuable information for future migration rate for rural areas. We use the same methodology in the last section, 4.4., to predict future change in migration rate at different horizons. To highlight the importance of the cointegration relation of house prices and ageing, we compare values of the adjusted R^2^ for change in migration rate implied by the error correction model and the adjusted R^2^ values from a growth rate specification without the cointegration residuals. We report results for house price growth in Table 6.

**Table 6:** Predictability evidence: Change in migration rate.

Change in Migration Rate Projection	Rural Areas		Urban Areas	
	With cointegration residuals	Without cointegration residuals	With cointegration residuals	Without cointegration residuals
*Move*_*jt*+1_	0.206 (6.71***)	-0.001 (0.69)	-0.027 (0.95)	-0.024 (0.30)
*Move*_*jt*+2_	0.278 (7.35***)	-0.007 (0.44)	-0.067 (0.71)	-0.037 (0.14)
*Move*_*jt*+3_	0.380 (7.73***)	0.336 (1.46)	0.072 (17.73***)	0.074 (2.44)
*Move*_*jt*+4_	0.545 (7.59***)	0.339 (2.62)	0.351 (12.29***)	0.089 (2.18)

This table reports the adjusted *R*^*2*^ for migration rate projections implied by the error correction autoregressive specification with cointegration residuals ($Move_{jt+i}-{Move}_{jt}=\gamma_{0}\Delta Aged65+\gamma_{1}\hat{\nu_{jt}}+\epsilon_{j,t}$) and the growth-rate model without cointegration residuals ($Move_{jt+i}-{Move}_{jt}=\gamma_{0}\Delta Aged65_{t}+\epsilon_{j,t}$) for rural and urban areas. One-year to four-year projections of house price growth are reported.

As shown in Table 6, we observe similar patterns of predictions in migration rate changes as in house price growth. The cointegration residuals account for more variations of future migration rate change than the growth rate model without cointegration residuals across all horizons. Also the cointegration residuals account for more variations of future migration rate change in rural areas than that in urban areas again across all horizons. Only projections of migration rate change in rural areas with cointegration residuals have significant model F-statistics. Results in Table 6 suggest that cointegration residuals contain valuable information about migration rate changes in one- to four-year horizons.

Overall our findings in this section highlight the importance of the cointegration residuals in providing information on migration rates. Our findings also support the 2011 Rural Regeneration Act as migration rate increase with equilibrium adjustments of short-term deviations of house prices.

## 5. General implementations

In this section we discuss how to implement our empirical cointegration-based framework into other countries or market settings. The key assumption is that at least one linear combination of house prices and ageing populations are difference-stationary, hence cointegrated. Economic theories are needed to support this assumption.

**Step 1.** Synchronization of frequencies of housing, ageing, and macroeconomic variables. Suppose we use {*P*_*ijt*_} for transaction price of property *i* in area *j* on date *t*, and {*G*_*jm*_} for ageing and {***X***_*jm*_} for demographic and macroeconomic variables for area *j* during period *m* (e.g. month-year). Since property transactions are sparse on average and of irregular time interval, we estimate an area-specific house price level $\left\{{\hat{P}}_{jm}\right\}$ from {*P*_*ijt*_} for area *j* in period *m* where *t* belongs (i.e. *t*∈*m*). Standard techniques of house price index construction could be employed to convert frequencies of property transactions into regular intervals. Section 3 describes a house price index construction using repeat-sales approaches.

**Step 2.** Tests of stationarity of time series. We perform augmented [62] unit root test with Engle and Granger (1987) confidence intervals. We estimate $\hat{\phi }$ from:

$$\Delta {\hat{P}}_{jm}=\phi {\hat{P}}_{j,m-1}+\sum_{k=1}^{n}\mu_{j,m-k}\Delta {\hat{P}}_{jm-k}+\epsilon_{jm}$$ (9)

A negative $\hat{\phi }$ and significant ADF statistics implies unit root and non-stationarity.

**Step 3.** Cointegration of housing price indices and ageing population. We estimate cointegration restrictions of housing price indices and ageing population by [63]:

$${\hat{P}}_{jm}=f(\Delta G_{jm};X_{jm},\alpha )+\nu_{jm}$$ (10)

$$\Delta \hat{\nu_{jm}}=\phi \hat{\nu_{jm-1}}+\sum_{k=1}^{n}\mu_{j,m-k}\Delta \hat{\nu_{jm-k}}+\epsilon_{j,t}$$ (11)

The functional form *f*(Δ*G*_*jm*_; ***X***_*jm*_, ***α***) represents the possible choices of long run equilibrium association between house prices and ageing population from an econometrician’s view, ***α*** is a vector of parameters defining *f*. Our paper employs a linear reduced form implied by life-cycle consumption theory. $\hat{\nu_{jm}}$ is the residual estimated from (10) and $\Delta \hat{\nu_{jm}}$ is its first difference. Cointegration is represented by the statistical significance of (some) parameters ***α***. An insignificant $\hat{\phi }$ implies stationarity of the residuals *ν*_*jm*_, hence the existence of cointegration restrictions between ${\hat{P}}_{jm}$ and *G*_*jm*_, house price indices and ageing variables. Matrix ***X*** allow researchers to incorporate spatial and intertemporal information, e.g. climate change risks, into the cointegration restrictions. Function *f* could be generalized to non-linear functional forms for more complex economic theories as long as properties of *f* satisfy the Granger Representation theorem [65, 66].

**Step 4.** Estimating error correction term. To estimate the magnitude of error correction of the cointegration restriction between house price indices and ageing variables, coefficient $\hat{\gamma_{1}}$ could be estimated from an error correction model $\Delta {\hat{P}}_{jm}=\gamma_{0}\Delta G_{jm}+\gamma_{1}\hat{\nu_{jm}}+\epsilon_{j,t}$, where $\hat{\nu_{jm}}$ could be retain from Step 3. A significant negative $\hat{\gamma_{1}}$ represents that ${\hat{P}}_{jm}$, house prices, converge to the long-run relation defined by *f* in (10).

## 6. Conclusion

How does the riskiness of ageing population change with house price dynamics of rural areas? Why do rural house prices increase faster than cities despite their ageing populations? We introduce an empirical, robust, cointegration-based framework to answer these questions. This framework is applicable to most ageing societies with serviceable data of house prices and population. To illustrate its effectiveness, we apply our framework with property transactions and ageing population data from rural and urban areas of Taiwan. The longevity and consistency of property transactions and macroeconomic data from Taiwan makes it suitable to showcase the long run cointegrating relation of housing and ageing. We also discuss how to implement our framework in more general settings in Section 5.

When house prices and ageing population are cointegrated, we show that short-run deviations from their cointegrated level forecast strongly predict future rural house prices at long horizons. Contrary to current literature, cointegration residuals are insignificant predicting future urban house prices. Cointegration residuals also predict significant positive migration rates in rural areas but not in urban areas. Our framework is pertinent to ageing societies with available housing and demographic data. Our evidence highlights the importance of cointegration-based long-run ageing risks for rural housing markets. Our study has implications beyond a solitary case study as there are many ageing societies that recently adopted rural revitalization programs akin to Taiwan. As such our findings could be useful references for Japan, Italy, Germany, among other ageing societies. In fact countries worldwide are reinventing their public investment strategies to combat economic, health and social issues in relation to ageing population. We recommend Taiwan and countries facing ageing population to (1) continue and expand their public funding for rural areas through rejuvenation programs, (2) promote collaborations between public and private sectors (e.g. universities and large corporations) to explore sustainable development of rural areas, (3) bringing back young adults to rural hometowns with new opportunities. Our study of cointegration between house prices and ageing population provides partially an economic foundation of such recommendations.

One limitation of our paper is the availability of housing data in a microeconomic, project-specific setting. As housing markets are local by nature, project-specific revitalization is an interesting dimension yet to be explored. Other interesting future directions our paper have yet to study include risks of climate change, cultural heterogeneity, and cross country cointegration restrictions between ageing and housing.

## Supporting information

S1 Table Annualized house price indices of 17 counties/municipalities in Taiwan. (CSV)

S2 Table Comparison of variables definition for index construction between value weighted arithmetic repeat sales method and geometric repeat sales methodology. (DOCX)

S3 Table Monthly house price indices of 17 counties/municipalities in Taiwan. (CSV)
