# Supplementary material for: Cointegrations in house price dynamics and ageing population risks
Source: PLoS One. 2024 Feb 12;19(2):e0296991. doi: 10.1371/journal.pone.0296991 (PMC10861090; doi:10.1371/journal.pone.0296991)
Supplement: S2 Table — (DOCX) [file pone.0296991.s002.docx]

Table A2. Comparison of Variables Definition for Index Construction between Value Weighted Arithmetic Repeat Sales Method and Geometric Repeat Sales Methodology.

| Variables | Value Weighted Arithmetic Repeat Sales Methodology | Geometric Repeat Sales Method |
| --- | --- | --- |
| $y_{i}$ | $P_{i0}$ if the first sale of house *i* is in period 0, and zero otherwise | $ln\left( \frac{P_{ik}}{P_{ih}} \right)$ |
| $x_{ij}$ | -1 multiply by the price of the first sale of house *i* if the time of the first sale was *j*, the price of the second sale of house *i* if the time of the second sale was *j*, equals zero otherwise | -1 if the time of the first sale of house *i* was *j*, 1 if the time of the second sale of house *i* was *j*, equals zero otherwise |
